# Gut Microbiota Patterns Associated with Colonization of Different *Clostridium difficile* Ribotypes

**DOI:** 10.1371/journal.pone.0058005

**Published:** 2013-02-28

**Authors:** Jure Skraban, Saso Dzeroski, Bernard Zenko, Domen Mongus, Simon Gangl, Maja Rupnik

**Affiliations:** 1 Faculty of Medicine, University of Maribor, Maribor, Slovenia; 2 Centre for Microbiology, Institute of Public Health Maribor, Maribor, Slovenia; 3 Centre of Excellence for Integrated Approaches in Chemistry and Biology of Proteins, Ljubljana, Slovenia; 4 Jozef Stefan Institute, Ljubljana, Slovenia; 5 Jozef Stefan International Postgraduate School, Ljubljana, Slovenia; 6 Faculty of Electrical Engineering and Computer Science, University of Maribor, Maribor, Slovenia; Institute Pasteur, France

## Abstract

*C. difficile* infection is associated with disturbed gut microbiota and changes in relative frequencies and abundance of individual bacterial taxons have been described. In this study we have analysed bacterial, fungal and archaeal microbiota by denaturing high pressure liquid chromatography (DHPLC) and with machine learning methods in 208 faecal samples from healthy volunteers and in routine samples with requested *C. difficile* testing. The latter were further divided according to stool consistency, *C. difficile* presence or absence and *C. difficile* ribotype (027 or non-027). Lower microbiota diversity was a common trait of all routine samples and not necessarily connected only to *C. difficile* colonisation. Differences between the healthy donors and *C. difficile* positive routine samples were detected in bacterial, fungal and archaeal components. *Bifidobacterium longum* was the single most important species associated with *C. difficile* negative samples. However, by machine learning approaches we have identified patterns of microbiota composition predictive for *C. difficile* colonization. Those patterns also differed between samples with *C. difficile* ribotype 027 and other *C. difficile* ribotypes. The results indicate that not only the presence of a single species/group is important but that certain combinations of gut microbes are associated with *C. difficile* carriage and that some ribotypes (027) might be associated with more disturbed microbiota than the others.

## Introduction

*Clostridium difficile* infection (CDI) is recognised as the leading cause of nosocomial intestinal infections, mainly in elderly hospitalised patients 1]. The onset of CDI has been linked to the use of broad-spectrum antibiotics, historically especially with clindamycin 2,3]. This implicated very early that the healthy gut microbiota has an important role in disease development. The importance of gut microbes is also supported by the high success rate (around 90%) of biotherapy (faecal transplantations) used in patients with severe cases of recurring CDI 4–6].

Several studies on gut microbiota changes associated with *C. difficile* colonization or infection have been published recently. Mice treated with clindamycin loose up to 90% of bacterial taxons in the cecum and are rapidly colonized with *C. difficile* 7]. Other molecular studies on the changes of gut microbiota during CDI have used16S rRNA clone libraries and showed lower bacterial microbiota diversity in patients with a recurring CDI 8]. The banding patterns on gradient gels connected specific bacterial groups to *C. difficile* colonisation in adults 9] and infants 10]. Another study used 454 sequencing to compare the composition of gut microbiota of *C. difficile* positive (20 asymptomatic and 2 diarrhoeal samples) and negative subjects (252 samples). They found no differences in the composition of faecal microbiota in asymptomatic *C. difficile* carries and healthy subjects. However, in *C. difficile* 027 ribotype positive subjects (n  =  2) with diarrhoea at the time of sampling faecal microbiota had a lower overall diversity at the genus level 11].

As described, different molecular approaches (including denaturing gradient gel electrophoresis (DGGE), 16S rRNA clone libraries and metagenomics) were used in studies on *C. difficile* and changes in microbiota. Here we have applied a simple and high-throughput DHPLC method (DHPLC - denaturing high pressure liquid chromatography) which separates 16S rDNA amplicons based on fragment size and sequence to analyse human faecal microbiota in the *C. difficile* positive and negative routine samples. It has been shown previously, that DHPLC offers a level of analysis, which is similar to gradient gels (DGGE) in terms of sensitivity and resolution, with the advantage of high automatisation and repeatability 12,13]. It has been used successfully in community profiling of microbiota of the urogenital tract 14], detecting changes in gut microbiota of patients during antimicrobial therapy 12], following the development of gut microbiota in infants 15], and in profiling various other complex communities from fermenter sludge, compost or soil 13].

Studies on the association of gut microbiota and *C. difficile* mostly describe the bacterial microbiota and very rarely fungi 16], while no studies have included archaea. With DHPLC we have analysed the bacterial, but also the fungal and archaeal gut microbiota in human faecal samples with requested *C. difficile* testing. With machine learning analysis of the collected data, we were able to show that certain patterns of microbiota composition are associated with *C. difficile* colonization and in particular with the PCR ribotype 027.

## Materials and Methods

### Collecting and storing of the faecal samples

Faecal samples with requested *C. difficile* testing (n  =  171) were obtained from two different routine laboratories. These samples were routinely collected in plastic containers and have been stored at 4°C and shipped to the study site within 48 h, where they have been immediately frozen at −80°C until further processing.

According to the results of the routine *C. difficile* testing, 105 faecal samples were *C. difficile* positive and 66 were *C. difficile* negative. A stool sample was described as non-diarrhoeal or diarrhoeal (has taken the shape of the container). No further clinical information was obtained for the samples.

Additional 37 samples were collected from healthy donors and stored as described above. None of the samples from healthy donors was diarrhoeic. The age of the donors ranged from 14 to 72 years. The gender distribution was 22 females and 15 males.

### **Ethics Statement**

The National Medical Ethics Committee of the Republic of Slovenia (NMEC) has approved the study (number 126/05/12). NMEC has also approved that written or verbal consent is not needed for routine samples and that only verbal informed consent is needed for healthy volunteers. Documenting was not requested. In the case of minor/children participant´s informed consent was obtained from the parents.

### Detection, isolation and ribotyping of *C. difficile* from faecal samples

In each of the two routine laboratories, *C. difficile* was detected by different methods. One laboratory performed the VIDAS ToxA/ToxB test and culturing on CLO selective plates (BioMerieux) after ethanol shock. The second laboratory used the molecular Cepheid Xpert *C. difficile* assay. From the samples tagged as *C. difficile* positive by the Cepheid Xpert *C. difficile* assay, *C. difficile* was isolated by enrichment in Oxoid *C. difficile* broth with Oxoid supplement SR0096E, lysozyme (5 mg/l) and sodium cholate (1%) as described previously 17]. The same enrichment was also used to test the presence of *C. difficile* in the faecal samples of the healthy volunteers.

All isolates were confirmed as *C. difficile* by the amplification of *cdd3*, located downstream from the pathogenicity locus (*PaLoc*) 17] and ribotyped with primers described by Bidet 18]. The profiles were analysed with the BioNumerics software. All isolates were also toxigenic as determined by toxinotyping (data not shown) 19].

### Isolation of the total DNA from faecal samples and amplification of the marker genes

The extraction of DNA was performed with the DNA Stool Mini Kit (Qiagen, Germany) after mechanical disruption with the SeptiFast Lyse Kit (Roche) on MagNA Lyser (speed 7000, 70 s; Roche). The DNA concentration and purity were checked with Nanodrop and 60 ng of faecal DNA was used for PCR amplification of bacterial, archaeal and fungal marker genes.

Primers and cycling conditions described by Domann 14] were used for amplification of the variable region V6–V8 of bacterial 16S rRNA genes. The correct amplicon size (470 bp) was checked by agarose gel electrophoresis. Heteroduplex DNA molecules were reduced by reconditioning as described 20]. Before loading onto the DHPLC column, the PCR products were purified with the PCR Purification Kit (Qiagen).

For the amplification of archaeal 16S rRNA genes, we have targeted the variable region V3 for which we used a modified forward primer PARCH340F 5′- CCCTACGGGGTGCAGCAG -3′ previously described by Ovreas 21] and designed a new reverse primer A780R 5′- TACCCGGGTATCTAATCCGGT -3′ which anneals to a conserved region about 420 bp downstream. The cycling conditions were 95°C for 7 min, followed by 35 cycles using 95°C for 40 s, 63°C for 30 s, 72°C for 30 s and the final extension at 72°C for 5 min. We have checked the amplicon size, reconditioning and purification as described above.

The fungal internal transcribed spacer region 2 (ITS2) was amplified using a nested PCR. For the first reaction, we have used the primer pair nu-SSU-0817F 5′-TTAGCATGGAATAATRRAATAGGA-3′ 22] and ITS4R-5′ TCCTCCGCTTATTGATATGC-3′ 23]. The cycling conditions were 95°C for 2 min, followed by 14 cycles using 95°C for 30 s, 61.3°C for 30 s with a 0.5°C decrement for each following cycle and 72°C for 60 s. This was followed by 19 cycles at 95°C for 30 s, 54.3°C for 30 s and 72°C for 60 s and the final extension at 72°C for 5 min. The correct size of the amplicons (1400–2000 bp) was checked by agarose gel electrophoresis. Before the second PCR reaction, we have degraded any remaining primers from the first PCR reaction. For the degradation, the Shrimp Alkaline Phosphatase and Exonuclease I (both from Fermentas) were used. The reaction mixture of the final volume of 6.5 µl contained 0.15 U/ µl of the Phosphatase, 1.5 U/ µl of the Exonuclease I and 5 µl of the PCR product from the first reaction. The mixture was incubated at 37°C for 15 min and then the enzymes deactivated (85°C, 15 min). Five µl were then used as the template for the second PCR reaction using the primer ITS86F 5′-GTGAATCATCGAATCTTTGAAC-3′ 24] and the ITS4R primer described above. The cycling conditions were 95°C for 2 min, followed by 25 cycles at 95°C for 30 s, 54.3°C for 30 s and 72°C for 40 s and the final extension at 72°C for 5 min. The right size of the amplicons (approximately 200–400 bp) was again checked on agarose gel and the products purified as described above.

### Separation of the amplified DNA with DHPLC

To separate the PCR products, we have used denaturing high pressure liquid chromatography (DHPLC; WAVE Microbial Analysis System; Transgenomics, USA). The amplicon volumes loaded on the column were 20 µl for bacteria, 15 µl for archaea and 10 µl for fungi. Conditions for separating the bacterial and fungal amplicons were described previously by Domann 14] and Goldenberg 25], respectively. For archaea, we have optimised the separation conditions which are given in the Supplementary table S1. A UV detector was used in DNA detection and the results were analysed with the Navigator Software version 2.2.0 (Build 25), the peak analysis parameters are given in the Supplementary table S1.

### Normalization of retention times and assigning the DHPLC peaks to microbial groups

Before analysis, the retention times of the DHPLC peaks were normalized. A marker designed as a G - C rich oligonucleotide, which eluted after the sample, was loaded onto the column together with the sample. The retention times of the peaks were then adjusted according to the equation 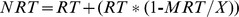, where NRT stands for the normalized retention time, RT for the retention time, MRT for the retention time of the marker and X for an arbitrarily chosen point on the retention time scale.

### Identification of the microbial groups

The retention time scale of the DHPLC profiles was divided into 0.2 min intervals. The representative peaks within each 0.2 min interval were collected, sequenced and all peaks within the given 0.2 min interval were then assigned to a bacterial group as described below.

The DHPLC peak fractions were collected form well separated and overlapping peaks and sent for sequencing (Eurofins MWG Operon, Germany). In case of partially overlapping peaks, the collected DHPLC peak fractions sometimes resulted in mixed sequence. In this case the corresponding PCR products were cloned into pGEM®-T Easy Vector System (Promega, Germany) following the manufactureŕs instructions. Ten colonies from each cloned fraction were collected, 16S rRNA gene fragments re-amplified (as above), purified (as above) and screened by DHPLC. Those, which differed in retention times, were selected and sequenced (Eurofins MWG Operon, Germany). The sequences were compared to the databases provided by the National Centre for Biotechnology Information (NCBI) and Ribosomal Database Project-Release 10 and the DHPLC peak fractions named accordingly. All 0.2 min intervals were named after the organisms identified in the fractions belonging to a particular interval. If more than one organism was identified in the DHPLC fractions taken within a particular 0.2 min interval, that interval was named after all the identified organisms. The named time intervals were from now on referred to as microbial groups.

### Statistical analysis

The 208 faecal samples were divided into seven sets of samples, based on the following criteria: healthy donors or routine samples, diarrhoea or formed stool, the presence or absence of *C. difficile*, and 027 ribotype or other ribotypes. The names of the sets and the number of samples in each set are given in Tables 1, 2 and 3.

**Table 1:** Comparison of routine *C. difficile* negative samples (set of samples with formed stool and set with unformed stool) vs. healthy donors by logistic regression model.

Sample set 1 vs. Sample set 2 ^a^	NEG-F (29) vs. Healthy (37)	NEG-D (37) vs. Healthy (37)
*Peptostreptococcaceae*		0.21 (0.0028)
*Ruminococcus bromii*		0.11 (0.0454)
*Bifidobacterium longum*	0.27 (0.0288)	
*Enterobacteriaceae* 3	0.25 (0.0088)	0.25 (0.0056)
*Faecalibacterium sp.*		0.08 (0.0201)
*Bacteroides uniformis*		0.29 (0.0161)
*Prevotella sp.*	0.23 (0.0256)	0.18 (0.007)
*Alistipes sp.*	0.2 (0.0133)	0.14 (0.0014)
*Escherichia*/*Shigella sp*.	0.17 (0.0012)	0.21 (0.0028)
*Bacteroides vulgatus*	0.11 (0.0059)	0.3 (0.0331)

a - the compared sample sets and the number of samples in each set is specified.

NEG-F: *C. difficile* negative/formed stool; NEG-D: *C. difficile* negative/diarrhoea; HEALTHY: healthy donors.

Results are presented as odds ratios for individual microbial groups between different sets of samples. An odds ratio below 1 indicates that this particular microbial group is less frequent in sample set 1 as compared to sample set 2. The odds ratios are given in the table only for the p-values (shown in parentheses) below 0.05 for the compared sample sets.

**Table 2:** Comparison of different sets of routine *C. difficile* positive samples vs. healthy donors by logistic regression model.

Sample set 1 vs. Sample set 2 ^a^	OTHER-F (17) vs. Healthy (37)	OTHER-D (31) vs. Healthy (37)	027-F (16) vs. Healthy (37)	027-D (41) vs. Healthy (37)
*Peptostreptococcaceae*			0.25 (0.0285)	
*Ruminococcus bromii*			0.05 (0.0069)	
*Bifidobacterium longum*	0.18 (0.0341)	0.25 (0.0197)	0.09 (0.0247)	0.03 (0.0013)
*Enterobacteriaceae* 1	0.07 (0.018)		0.06 (0.0149)	0.1 (0.0323)
*Bacteroides sp*. 1	0.3 (0.0461)	0.35 (0.0385)	0.19 (0.011)	0.22 (0.0019)
*Enterobacteriaceae* 2		0.26 (0.0116)	0.15 (0.0036)	0.13 (0.0001)
*Enterobacteriaceae* 3		0.2 (0.0021)	0.07 (0.0013)	0.08 (0.0000)
*Bacteroides sp.* 2		0.28 (0.0326)	0.1 (0.0322)	0.12 (0.0017)
*Faecalibacterium sp.*	0.04 (0.0042)			0.1 (0.0323)
*Bacteroides uniformis*		0.35 (0.0403)	0.12 (0.0106)	0.27 (0.0084)
*Prevotella sp.*	0.17 (0.0153)	0.22 (0.0198)	0.12 (0.0038)	0.15 (0.0025)
*Alistipes sp.*	0.11 (0.0019)	0.19 (0.0105)		0.14 (0.0014)
*Escherichia*/*Shigella sp*.	0.13 (0.0022)	0.09 (0.0000)	0.19 (0.0105)	0.15 (0.0002)
*Bacteroides vulgatus*		0.1 (0.0044)		0.12 (0.0017)
*Candida albicans*				3.62 (0.0115)
*Candida glabrata*				10.45 (0.0301)
*Methanobrevibacter smithii*	0.23 (0.0189)	0.20 (0.0025)		0.25 (0.0041)

a - the compared sample sets and the number of samples in each set is specified.

OTHER-F: *C. difficile* non 027 ribotype/formed stool; OTHER-D: *C. difficile* non 027 ribotype/diarrhoea; 027-F: *C. difficile* 027 ribotype/formed stool; 027-D: *C. difficile* 027 ribotype/diarrhoea; HEALTHY: healthy donors.

Results are presented as odds ratios for individual microbial groups between different sets of samples. An odds ratio below 1 indicates that this particular microbial group is less frequent in sample set 1 as compared to sample set 2. The odds ratios are given in the table only for the p-values (shown in parentheses) below 0.05 for the compared sample sets.

**Table 3:** Comparisons of all routine *C. difficile* positive vs. all routine *C. difficile* negative samples by logistic regression model.

Sample set 1 vs. Sample set 2 ^a^	OTHER-D (31) vs. NEG-D (37)	027-D (41) vs. NEG-D (37)	027-D (41)/OTHER-D (31) vs. NEG-D (37)
*Streptococcus sp*./*Enterococcus sp*. 2		3.7 (0.0471)	3.23 (0.0461)
*Peptostreptococcaceae*		2.9 (0.0259)	2.63 (0.0232)
*Ruminococcus bromii*		4.8 (0.0491)	3.33 (0.0453)
*Bifidobacterium longum*		0.04 (0.0031)	0.12 (0.0012)
*Enterobacteriaceae* 1		0.1 (0.037)	
*Bacteroides sp*. 1		0.39 (0.0465)	
*Enterobacteriaceae* 2		0.35 (0.0289)	
*Enterobacteriaceae* 3		0.33 (0.0498)	
*Methanobrevibacter smithii*	0.39 (0.0491)	0.31 (0.0268)	0.35 (0.0138)

a - the compared sample sets and the number of samples in each set is specified.

NEG-D: *C. difficile* negative/diarrhoea; OTHER-D: *C. difficile* non 027 ribotype/diarrhoea; 027-D: *C. difficile* 027 ribotype/diarrhoea.

Results are presented as odds ratios for individual microbial groups between different sets of samples. An odds ratio below 1 indicates that this particular microbial group is less frequent in sample set 1 as compared to sample set 2. The odds ratios are given in the table only for the p-values (shown in parentheses) below 0.05 for the compared sample sets.

The results were analysed by looking at the differences between the sets of samples in terms of presence or absence of microbial groups or by looking at the percentage of peak area within the total analysed chromatogram area which was computed by the Navigator Software version 2.2.0 (Build 25) using parameters as described in the Supplementary table S1.

The difference in the number of organism groups was analysed with ANOVA. The equality of the variances in each group was first verified with the Levene test and the pre -planned post-hoc comparisons were carried out with contrast analysis. Since the observed contrasts were not orthogonal, the p-values were adjusted with the Holm method to control the type I error.

Diversity of microbiota was assessed with Reciprocal Simpsońs index of diversity and the differences between the compared sets of samples evaluated with Student T-test.

Logistic regression was used to analyse the presence or absence of individual microbial groups. The dependency between the dependent variable (1- individual has an organism group, 0 - individual does not have an organism group) and a microbial group was first tested with the chi - square test. After logistic regression, we used a contrast analysis to carry out the pre-planned comparisons between the sets of samples.

### Principal component analysis

Principal Component Analysis (PCA) with respect to Instrumental Variables was performed with R programme (version 2.15.1) and applications ade4 (version 1.5–0) and ade4TkGUI (version 0.2–5). We used the data matrix based on the presence of the peak and percent area of the peaks (representing an individual microbial group) from the DHPLC chromatograms to calculate the spatial coordinates of each faecal sample. We also calculated the centre of gravity and dispersion for each set of samples. Statistical significance of PCA clustering based on microbiota profiles was assessed using a Wilcoxon signed-rank test.

### Machine learning analysis

We further analysed the differences between the sets of samples in terms of presence or absence of microbial groups by using machine learning methods for the induction of decision trees 26], more specifically classification trees 27]. In particular, we used the WEKA J48 implementation 28] of the C4.5 algorithm 26]. The default parameter settings for J48 were applied, which include a minimum of two examples per leaf for pre-pruning and a confidence level of 0.25 for post-pruning.

The dependent (class) variable in this analysis was the ribotype of *C. difficile* detected in a sample (none, 027, other). The independent variables (attributes) were the indicators of presence/absence of each of the 34 microbial groups, as well as an indicator of the sample category (healthy donor/routine sample).

We next reversed the direction of analysis and analysed the overall microbiota composition depending on the detected ribotype of *C. difficile* and other sample properties. To this end, we used predictive clustering trees - PCTs 29] for multi-target classification, as implemented in the Clus data mining suite (http://clus.sourceforge.net). Each of the indicators of presence/absence of the 34 microbial groups was considered as a target (class) variable, all of them to be predicted simultaneously by a single tree. As independent variables (attributes), we considered the sample category (healthy donor/routine sample), the consistency of the stool (formed/diarrhoea) and the ribotype of *C. difficile* detected in a sample (none, 027, other).

## Results

The aim of this study was to compare the gut microbiota in faecal samples from healthy donors and from routine samples negative or positive for *C. difficile* and to detect possible differences in dominant microbial groups (bacteria, archaea, fungi).

We have analysed 208 faecal samples, of which 171 were routine samples and 37 were from healthy volunteers. All faecal samples from healthy volunteers were *C. difficile* negative. Of the 171 routine samples, 105 were *C. difficile* positive and 66 were *C. difficile* negative. From all 105 positive faecal samples *C. difficile* was isolated and strains were assigned to 22 different *C. difficile* PCR ribotypes. The five most frequent ribotypes were 027 (57), 014/020 (6), 081 (6), 002 (5) and 023 (5).

### DHPLC analysis of archaeal, fungal and bacterial microbiota

**Figure 1:**
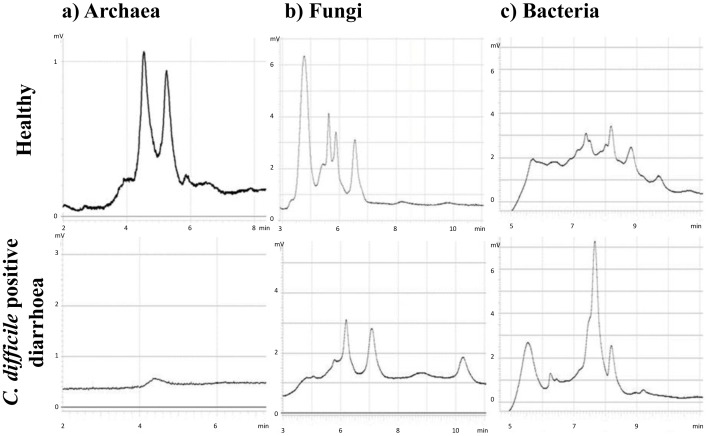
Representative DHPLC profiles of faecal microbiota. The profiles show archaeal (a), fungal (b) and bacterial (c) faecal microbiota. The faecal profiles of healthy volunteers are in the upper row and the profiles from the diarrhoeal patients colonised by *Clostridium difficile* 027 ribotypes are in the lower row. The selected profiles show typical chromatograms of the three studied microbial groups. The chromatograms show increasing complexities from archaeal to fungal and bacterial microbiota, respectively.

The representative DHPLC profiles in Fig. 1 show different complexity of archaeal, fungal and bacterial microbiota in human faecal samples. This section will only briefly list archaeal, fungal and bacterial groups detected by the DHPLC method, while the differences in faecal microbiota between samples (healthy, *C. difficile* positive and negative samples) will be described in more detail in further sections.

The archaeal microbiota was simple in all 208 analysed samples and was composed maximally of two methanogenic archaea, *Methanosphaera stadtmanae* and *Methanobrevibacter smithii* (Fig. 1a).

**Figure 2:**
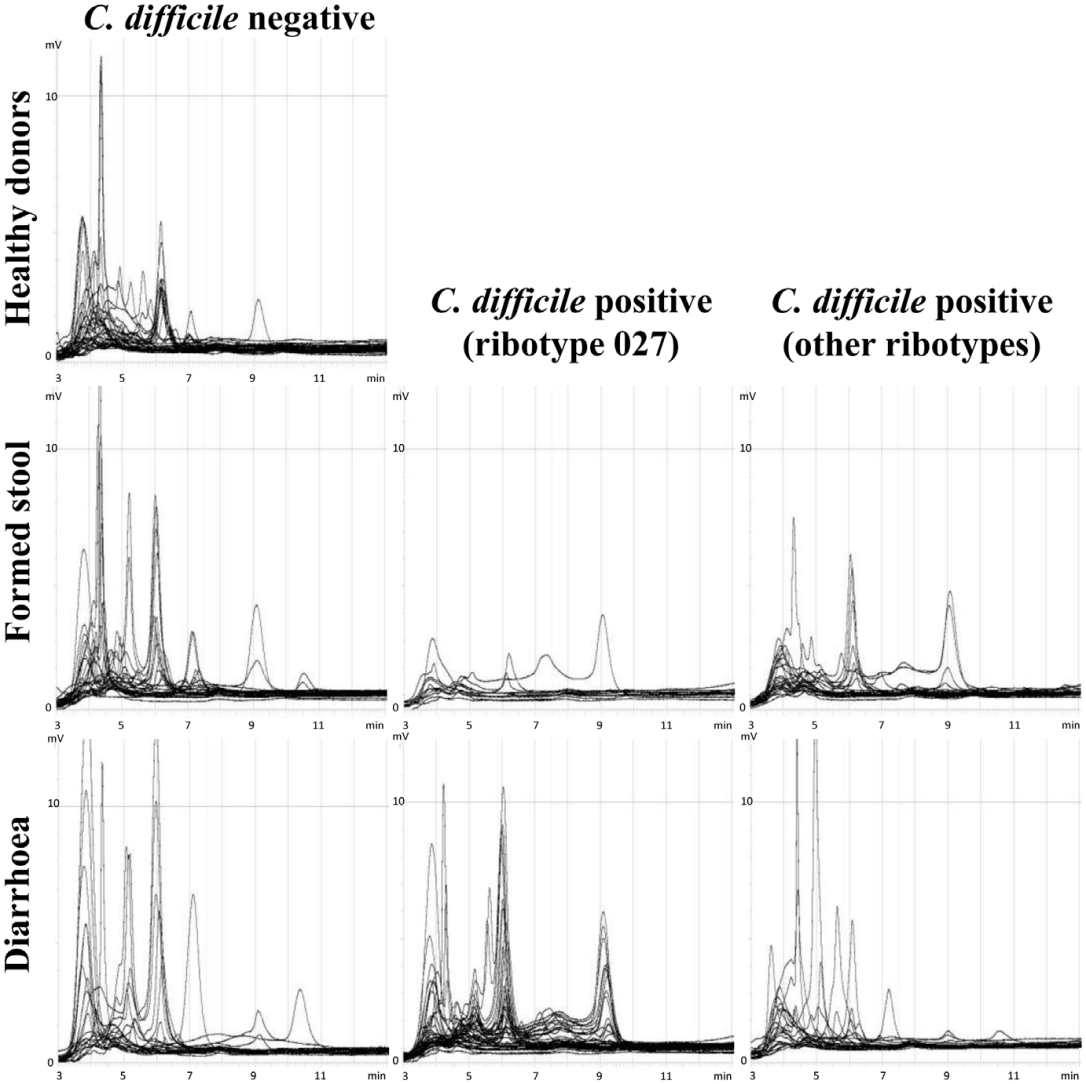
Fungal DHPLC profiles of all seven sets of samples. The samples were distributed into sets according to consistency, source (healthy/routine) and *C. difficile* presence. The peaks with different retention times (x - axis) represents different fungal groups.Fungal microbiota in healthy volunteers and *C. difficile* positive samples differs in the composition (position of the peaks) but not in diversity (number of the peaks).

The fungal microbiota was more diverse (Fig 1b, Fig. 2). We have found nine different fungi altogether. The most frequent among them were *Saccharomyces cerevisiae* (in 57% of samples) and *Candida albicans* (in 27% of samples). *Candida glabrata*, *Candida diversa*, *Clavispora lusitaniae*, *Pichia burtonii*, *Lemoniera sp*., *Eurotium sp*. and *Scleroderma sp*. were found only sporadically.

**Figure 3:**
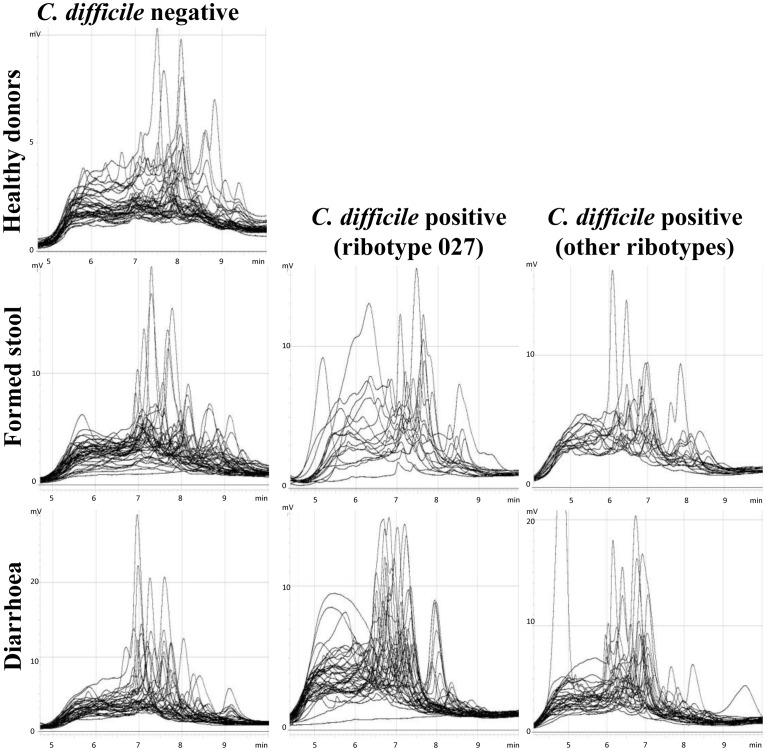
Bacterial DHPLC profiles of all seven sets of samples. Bacterial microbiota in healthy individuals is composed of a large number of species, hence the peaks are numerous but the area below each peak is small. The presence of *C. difficile* correlates with decreased diversity reflected by a lower number of peaks which are, however, more prominent.

Bacterial microbiota was most complex (Fig. 1c, Fig. 3). Altogether we have identified 23 different bacterial groups. Some chromatographic peaks were assigned to more than one genus because they either contained mixed sequences, or because the short sequences used in the DHPLC analysis did not allow distinction between them (e.g. *Clostridium difficile*/*Sporacetigenium sp*.; *Citrobacter freundii*/*Enterobacter sp*.; *Streptococcus sp*./*Enterococcus sp*. 1 and 2). Bacterial groups that were identified to the same genus or family but had different retention times were designated by a number (*Enterobacteriaceae* 1, 2 and 3; *Bacteroides sp*. 1 and 2, *Streptococcus sp*./*Enterococcus sp*. 1 and 2). Because short fragments are used in the DHPLC analysis further identification of particular amplified fraction was not possible. The remaining identified groups were *Clostridium sp*., *Peptostreptococcaceae*, *Blautia sp*., *Ruminococcus bromii*, *Ruminococcus sp*., *Faecalibacterium sp*., *Klebsiella pneumonia*; *Escherichia/Shigella sp*., *Prevotella sp*., *Alistipes sp*., *Bacteroides uniformis*, *Bacteroides vulgatus*, *Bifidobacterium longum* and *Bifidobacterium sp*.

### DHPLC profiles and principal component analysis

All 208 faecal samples were partitioned into seven sets according to origin (routine sample/healthy), appearance (diarrhoea/formed) and *C. difficile* colonization status (negative/ribotype 027/any other ribotype). We have named the sample sets accordingly (diarrhoeal/*C. difficile* negative samples; diarrhoeal/*C. difficile* ribotype 027 positive samples; diarrhoeal/*C. difficile* other ribotypes positive samples; formed stool/*C. difficile* negative samples; formed stool/*C. difficile* ribotype 027 positive samples; formed stool/*C. difficile* other ribotypes positive samples; healthy donors) (Fig. 2 and 3). Because of the large number of samples colonised with the 027 ribotype (57 out of 105 colonised samples), we were able to separate those from other ribotypes and treat it as a separate set of samples.

**Figure 4:**
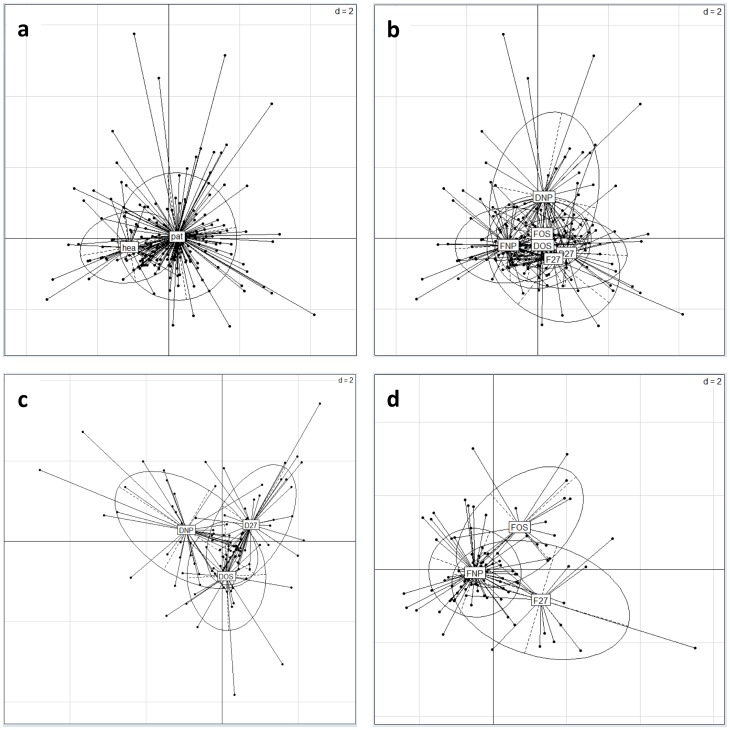
Principal component analysis of DHPLC profiles of dominant bacterial, fungal and archaeal species. Individual samples (represented by black dots) were clustered into sets according to *C. difficile* presence and type, origin and consistency (elipses) and the centre of gravity computed for each set of samples. All sets of samples show large dispersion and overlap (elipses). Figure 4a compares the samples sent for routine *C.difficile* testing (pat) and healthy donors (hea). Particularly the former set shows a great dispersion. Figures 4b, c and d compare different sets of samples from the patients. Figure 4b shows comparison of six different sets of routine samples; a greater shift in the centre of gravity between the formed and the diarrhoeal *C. difficile* negative samples (FNP/DNP), compared to the *C. difficile* positive samples (FOS/DOS, F027/D027). Fig 4c shows only the subset of different routine diarrheic samples. Fig 4d shows only the subset of different routine formed samples DNP - diarrhoeal/*C. difficile* negative samples, D27 - diarrhoeal/*C. difficile* ribotype 027 positive samples, DOS–diarrhoeal/*C. difficile* other ribotypes positive samples, FNP - formed stool/*C. difficile* negative samples, F27 - formed stool/*C. difficile* ribotype 027 positive samples, FOS - formed stool/*C. difficile* other ribotypes positive samples, pat - samples sent for routine *C. difficile* testing, hea - healthy donors).

To compare the faecal profiles of the dominant microbiota in the seven sets of samples, we first used PCA to calculate the spatial coordinates of each sample (Fig. 4). All sets of samples showed a large dispersion, particularly samples sent for routine *C. difficile* testing (Fig. 4a). Although the centres of gravity were different for samples from healthy persons and for routine samples, they also substantially overlapped (Fig. 4a). The result was similar when we compared the samples based on appearance (diarrhoea/formed) and *C. difficile* colonisation status (Fig. 4b, c and d).

Regardless of the overlap between the sets of samples, Wilcoxon signed-rank test performed on each pair of the sets from Fig. 4 (healthy donors or routine samples, diarrhoea or formed stool, the presence or absence of *C. difficile*, and 027 ribotype or other ribotypes) show a clear distinction between them. In all the cases, location shift greater than 0.5 can be shown with 95% confidence, while the probability of sets being different is larger than 99.9%. This confirms that the partition of the samples into the sets at hand was appropriate and we kept this grouping to further analyse the differences in microbial groups using statistical analyses and machine learning methods.

### Comparison of microbiota present in routine samples and in samples from healthy volunteers

**Figure 5:**
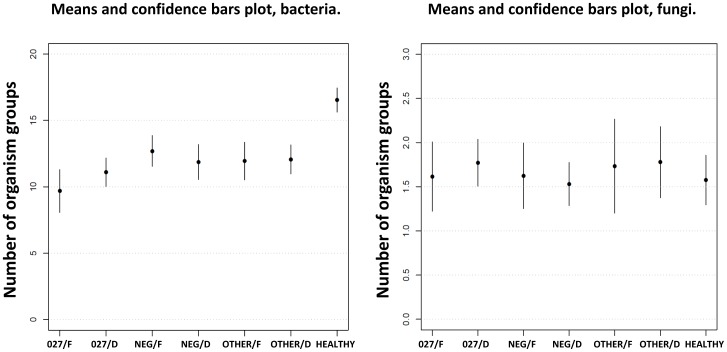
Average numbers of bacterial and fungal groups in different sets of faecal samples. The average numbers of present bacterial and fungal groups within each sample set are presented as means with confidence bars plot. 027/F: *C. difficile* 027 ribotype/formed stool; 027/D: *C. difficile* 027 ribotype/diarrhoea; NEG/F: *C. difficile* negative/formed stool; NEG/D: *C. difficile* negative/diarrhoea; OTHER/F: *C. difficile* non 027 ribotype/formed stool; OTHER/D: *C. difficile* non 027 ribotype; HEALTHY: healthy donors.

Within each of the seven sample sets, we have determined the average number of bacterial or fungal groups (Fig. 5). The difference in the average number of present bacterial groups between the healthy volunteers and each of the six different routine sample sets was statistically significant (P<0.0001). The comparison of the average number of bacterial and fungal groups shows that the healthy donors had the highest number of bacterial groups (in average 16.54) while the highest average among the routine samples 12.69 was the one for formed/*C. difficile* negative stools. The lowest average number of bacterial groups was observed in the samples colonised with *C.difficile* ribotype 027 (9.69 and 11.10 for formed and diarrhoeal stools respectively). The fungal microbiota showed no difference in the average number of fungi between the healthy donors and routine samples (Fig. 5). Simpsońs reciprocal index of diversity showed similar differences between the sets of samples with healthy donors having significantly higher microbiota diversity (10.03) than routine samples (from 4.47 to 8.38). In addition, the samples colonised with the ribotype 027 had significantly lower diversity (4.47 and 5.37, formed and diarrhoeal samples respectively) than samples colonised with other *C. difficile* ribotypes (6.25 and 8.38, formed and diarrhoeal samples respectively). The diversity indexes and P-values are given in Supplementary table S2.

After assessing differences between the average numbers of microbial groups, we have further analysed the differences on the level of specific microbial taxons with PCT (predictive clustering trees) analysis and with logistic regression. Several PCTs were built, relating microbiota composition to different combinations of independent variables. Here we present the trees built using the sample category only (Table 4) and the ribotype of *C. difficile* only (Table 5). The PCT models take into account only microbial groups present at high frequencies (above 50% in at least one sample set). We highlight the differences between healthy donors and routine samples (Table 4) and between routine samples with different *C. difficile* colonisation/ribotype status (Table 5), and only list the microbial groups that are present differentially (i.e., present in the majority of one set of samples and absent from the majority of the other set of samples). When healthy donors were compared to all routine samples (not distributed into sets regarding stool consistency and *C. difficile* presence) by PCT, five microbial groups were identified to be more frequent in healthy donors, including different *Enterobacteriaceae* and *Bacteroides sp*. (Table 4).

**Table 4:** Differences in microbial groups between healthy donors and routine samples as detected by the PCT model.

Organism group	Healthy donor	Routine samples
*Enterobacteriaceae* 2	1	0
*Enterobacteriaceae* 3	1	0
*Escherichia/Shigella sp*.	1	0
*Bacteroides sp*.1	1	0
*Bacteroides uniformis*	1	0

An entry of 1 means that the organism group (row) was present and an entry of 0 means that the organism group was absent in the majority of samples of the considered type (column).

**Table 5:** Differences in microbial groups present between different types of *C. difficile* colonisation as predicted by the PCT model.

Organism group	*C. difficile* **negative**	*C. difficile* **non 027 ribotype**	*C. difficile* **027 ribotype**
*Enterobacteriaceae* 2	1	1	0
*Bacteroides sp*. 1	1	0	0
*Escherichia/Shigella sp*.	1	0	0
*Saccharomyces cerevisiae*	1	1	0
*Methanobrevibacter smithii*	1	0	0

An entry of 1 means that the organism group (row) was present and an entry of 0 means that the organism group was absent in the majority of samples of the considered type (column)

Subsequently, all microbial groups were analysed by logistic regression to calculate the odds ratios for individual microbial groups when comparing sets of samples one to another (Tables 1, 2 and 3). In logistic regression, the routine samples were also further distributed into sets (*C. difficile* negative, 027 *C. difficile* positive, other than 027 *C. difficile* positive). Healthy donor microbiota differed from *C. difficile* negative routine samples mainly in bacterial composition (Table 1). On the other hand, differences between the healthy donors and *C. difficile* positive routine samples were detected in bacterial, fungal and archaeal components (Table 2).

### Changes of microbiota associated with *Clostridium difficile* presence in the faecal sample

As described before the average number of bacterial and fungal groups was not significantly different between the routine samples with a different *C. difficile* colonisation status and ranged from 9.69 bacterial groups in diarrhoeal/*C. difficile* ribotype 027 positive and to 12.69 bacterial groups in formed/*C. difficile* negative samples, respectively, and (on average) from 1.5 to 2.0 fungal groups per sample (Fig. 5).

When different subsets within the routine samples were compared by the PCT model, which takes into account only microbial groups present at high frequencies (above 50% in at least one sample set), we identified five microbial groups characteristically present or absent (the majority of) in different groups of routine samples (Table 5). All five were predicted to be present in *C. difficile* negative routine samples and only two (*Enterobacteriaceae* group and *S. cerevisiae*) in routine samples colonized with *C. difficile* ribotype other than 027.

When comparing *C. difficile* negative or positive samples to healthy volunteers, bacteria and archaea were less frequent in routine samples than in healthy donors, while some fungi were more frequent in *C. difficile* positive samples (Tables 1 and 2).

When comparing diarrhoeal *C. difficile* negative samples to diarrhoeal *C. difficile* positive samples (regardless of the ribotype) significant differences were found in four bacterial groups (*Peptostreptococcaceae*, *Ruminococcus bromii*, *Streptococcus/Enterococcus sp*. 2 and *Bifidobacterium longum*) and one archaeal group (*Methanobrevibacter smithii*) (Table 3, last column). Among them, *Bifidobacterium longum* and *Methanobrevibacter smithii* showed lower frequencies in the *C. difficile* positive samples. However, these differences were mainly due to the diarrhoeal 027 positive *C. difficile* samples as it will be described below.

Archaea (*Methanobrevibacter smithii*/*Methanosphaera stadtmanae*) were associated with healthy donors (detected in 65% (*M. smithii*)/22% (*M. stadtmanae*) of samples) and *C. difficile* negative samples (52%/21% for formed stool and 51%/19% for diarrhoea). *C. difficile* positive samples (especially those colonized with non - 027 ribotype) were characterised by lower frequencies of archaea. Frequencies of *M. smithii*/*M. stadtmanae* in 027 positive *C. difficile* samples were (38%/19% for formed stool and 32%/17% for diarrhoea) and in non - 027 positive samples (29%/12% for formed stool and 26%/10% for diarrhoea). The diarrhoeal non - 027 positive *C. difficile* samples and diarrhoeal *C. difficile* negative samples significantly differed to each other only in *Methanobrevibacter smithii* (Table 3, first column).

**Figure 6:**
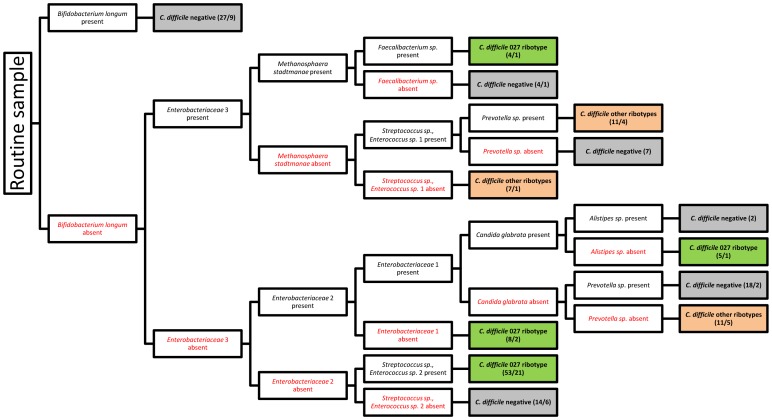
J48 decision tree describing the patterns of colonisation associated with presence or absence of *C. difficile*. Decision tree is showing only routine sample sets. The decision tree shows combinations of microbial groups that predict the outcome regarding *C. difficile* colonisation. The numbers in parentheses show the total number of samples and the number of incorrectly cassified instances, respectively.

A decision tree was constructed by the J48 machine learning approach, based on the presence or absence of microbial groups, to predict the outcome regarding *C. difficile* colonisation. The resulting decision tree splits the samples by category first. We only discuss the part that refers to the routine samples, shown in Figure 6. This decision tree was built automatically from the data on the collected samples. Each internal node is labeled with a test on the presence/absence of a microbial group, while each terminal node (leaf) is labeled with the most frequent class/outcome (*C. difficile* 027/*C.difficile* other ribotypes/*C. difficile* negative). The numbers in parentheses provide an indication of the accuracy of the prediction made by the leaf (total number of samples in the leaf/incorrectly classified samples). The tree therefore summarizes the data on the samples and the results of their analysis, relating microbial group composition to the outcome.

The decision tree can also be used as a predictive model, i.e., to make predictions regarding the *C. difficile* ribotype of new samples based on their microbial groups. In this case, its accuracy would have to be estimated on unseen data (and not on the training data as done here) by using procedures such as cross-validation.

The tree identifies several different combinations of microbial groups associated with the same outcome. The presence as well as absence of microbial groups influenced the outcome importantly. The microbial groups identified by the model to be most related to the outcome were bacteria, but also fungi and archaea (Fig. 6). The most strongly related microbial group was *Bifidobacterium longum* the presence of which was linked to the *C. difficile* negative outcome in 18 out of 27 cases. The presence of *Prevotella sp*. was associated with both, *C. difficile* positive and negative status, depending on the presence or absence of other microorganisms. It was associated with *C. difficile* absence when found together with *Enterobacteriaceae* 1 and 2. This was also the pattern with the best classification accuracy, misclassifying only 2 out of 18 samples. On the other hand, *Prevotella sp*. was associated with *C. difficile* presence when found together with *Enterobacteriaceae* 3 and *Streptococcus sp*./*Enterococcus sp*. 1.

### Specific changes in microbiota in the patients colonised with the *C. difficile* ribotype 027

Because a large number of *C. difficile* positive routine samples had ribotype 027 (57/105) we could study those samples as a separate set. We found most differences (8 bacterial groups) between the diarrhoeal *C. difficile* negative and diarrhoeal ribotype 027 positive samples (Table 3). Three bacterial groups (*Peptostreptococcaceae*, *Ruminococcus bromii* and *Streptococcus/Enterococcus sp*. 2) were more frequent and five bacterial groups (*Enterobacteriaceae* 1, 2, 3; *Bacteroides sp*. 1 and *Bifidobacterium longum*) were less frequent in *C. difficile* ribotype 027 positive samples. Among them *Bifidobacterium longum* had the highest statistical significance (P = 0.0031) (Table 3).

The analysis of the fungal groups showed that the diarrhoeal samples positive for the *C. difficile* ribotype 027 were more frequently colonised by *Candida albicans* and *Candida glabrata*, compared to stools from healthy donors (Table 2). However, no differences in fungi were detected among diarrhoeal samples positive for the *C. difficile* ribotype 027 and other diarrhoeal routine samples.

The machine learning approach identified four colonization patterns associated with the 027 ribotype (Fig. 6). They are different one to another and include bacteria, fungi and archaea. The pattern with the best predictive score, albeit associated with small number of samples (1 misclassified sample out of 5), involves the presence of *Enterobacteriaceae* 1 and 2 as well as *Candida glabrata*. In addition to the logistic regression analysis (Tables 2 and 3) and the J48 model (Fig. 6), specific changes in the samples colonised by the *C. difficile* ribotype 027 were detected also by the PCT model (Table 5). Table 5 shows only those five microbial groups for which the predicted outcomes between the sample sets were different. All of them are predicted to be present in *C. difficile* negative routine samples, but their absence is predicted in *C. difficile* 027 positive samples.

## Discussion

In this study we have used the DHPLC method to detect changes in faecal microbial communities during *C. difficile* colonisation. Although the method is simpler than more laborious sequencing approaches and can detect only a small proportion of present microbial groups, we have shown here that it does detect differences between selected groups of faecal samples and that detected differences are in agreement with studies published to date.

The tested faecal samples were first distributed into those collected from healthy volunteers and routine samples with requested *C. difficile* testing. The latter were further divided into six sets according to stool consistency, *C. difficile* presence or absence and ribotype (027 or non-027) (Fig. 2 and 3).

In current study lower diversity was a common trait of routine samples (formed or unformed) and not necessarily connected only to *C. difficile* colonisation as average number of bacterial groups in all routine samples was significantly lower compared to healthy donors. Although 66 samples out of 171 routine samples were negative for *C. difficile*, patients did have changed microbiota (Fig. 5). The fact that samples were send to the routine testing could be either due to diarrhoea (with *C. difficile* risk factors), while for formed routine samples the reason for test requirement could be untypical abdominal discomfort or retesting after CDI treatment. No clinical data were collected to confirm this possible explanation. Lower bacterial diversity was previously specifically attributed to different gastrointestinal diseases. In addition to patients with a recurrent 8] or active CDI 11], lower bacterial diversity was also observed in patients suffering from Crohńs disease (CD) 30], diarrhoea-predominant irritable bowel syndrome 31] and chemotherapy 32]. In addition, antibiotic treatment has been shown to cause short-term 33] and long-term 34] shifts and decreased diversity in gut microbiota.

Our results are in concordance with the previous findings at the level of individual bacterial taxons which showed lower levels of *Bifidobacterium longum, Prevotella sp*. and *Bacteroides sp*.in *C. difficile* positive samples 11,35,36]. In addition, in routine samples *Bifidobacterium longum* proved to be the most important predictor for the *C. difficile* negative status. Bacterial groups, which in our study were more frequent in *C. difficile* positive samples, include *Ruminococcus bromii*, the family *Peptostreptococcaceae* and *Streptococcus sp*./*Enterococcus sp*. 2. The first two groups confirm the previous studies which showed the increased numbers of firmicutes 36], particularly *Ruminococcus sp*. in symptomatic adults 11] and asymptomatic infants 37]; and increased levels of *Clostridium sp*. in *C. difficile* colonised subjects 8,35]. In our study, significant increase in colonisation frequencies of *Streptococcus sp.*/*Enterococcus sp*. 2 group were found in diarrhoeal *C. difficile* colonised samples. Previous studies found a positive correlation between a persistent asymptomatic *C. difficile* colonization and enterococci 38] and connected vancomycin resistant enterococcus (VRE) infection to more than 50% of *C. difficile* colonisations which prompted the autors to suggest an active surveilance for VRE in *C. difficile* colonised patients 39].

Very few studies have so far reported on the fungal microbiota in connection to *C. difficile* colonization or the gut microbiota in general. In *C. difficile* colonized hamsters treatment with monoclonal antibodies against *C. difficile* resulted in proliferation of fungus *Wickerhamomyces sp*. 16]. In humans we have here described several fungi to be found in healthy or disturbed faecal microbiota. However, average number of fungal groups in faeces was low (1.5–2.0), which is similar to a previous study of fungal gut microbiota in faeces of healthy individuals reporting 1–3 fungal species per faecal sample 40]. Another culture independent study which investigated fungal diversity in patients with inflammatory bowel disease (IBD), showed a slightly higher number of mucosa-associated fungi (6 per patient) 41]. Comparison of diversity in patients with different types of intestinal inflammation (Crohńs disease (CD), ulcerative colitis, non-IBD colitis, unclear diarrhoea) showed, that only CD patients had a higher fungal diversity 41]. We did not find differences in fungal diversity between all *C. difficile* colonized and all non-colonized subjects. However, a specific subgroup of diarrhoeal patients, colonised with the 027 ribotype, was more frequently colonised with *Candida albicans* and *Candida glabrata* compared to the healthy donors. Our results suggest that while fungal diversity does not increase during CDI, there is a qualitative shift in the composition towards opportunistic pathogens.

This is the first study to look at the possible changes in archaeal microbiota in connection to *C. difficile* colonization. We found higher frequencies of a single methanogenic species in the healthy donors compared to *C. difficile* positive samples, but also when comparing the *C. difficile* negative samples, sent for the routine *C. difficile* testing, to the *C. difficile* positive samples. Our results are therefore linking *Methanobrevibacter smithii* to the microbiota of healthy individuals. Until recently, archaea have been a neglected part of gut microbiota studies. The importance of archaea for the homeostasis of the gut ecosystem and the role they play in the bacterial degradation of sugars has led to the increased studies of these microorganisms 42,43]. They have been implicated in obesity after gastric bypass surgery 44] and in periodontal diseases 45].Because of the large number of samples colonised with the ribotype 027, we could analyse them separately. Similar as observed before on a much smaller set of samples 11], our results also show that bacterial diversity in faecal samples colonized with *C. difficile* 027 was lower than in other *C. difficile* positive samples. Additionally, we showed that also certain fungi and archaea were absent in faecal samples positive for *C. difficile* 027. Although so called hypervirulence of ribotype 027 has to be considered with caution 46], it seems that this particular strain has increased potential to spread in the environment and to colonize the gut. One of the possible explanations for this would be superior colonizing properties of 027 strains and hence reduced need for an imbalanced microbiota. However, the association of ribotype 027 strains with the lowest microbiota diversity among all samples in this study potentially suggest that ribotype 027 is capable of actively affecting the composition of faecal microbiota and is therefore increasing its own colonization potential.

Current studies have characterised *C. difficile* specific changes in microbiota by evaluating relative frequencies and abundances for each gut microorganism separately. Here, in contrast, we have applied machine learning methods, and could show that *C. difficile* colonisation is associated with patterns in the gut microbiota composition. Some species are very strong predictors, as for example, *Bifidobacterium longum*, which was connected to the *C. difficile* negative outcome. Some other bacterial groups (e.g. *Prevotella*) could be associated with a *C. difficile* positive or negative status, which depends on the other microorganisms present in the microbiota. This indicates that not only the presence or absence of a single species/taxon but rather that certain combinations of gut microbes are important in *C. difficile* associated changes of microbiota.

## Supporting Information

Table S1 **The DHPLC separation conditions and peak analysis parameters.** Conditions for the separation of mixed species 16S rDNA or ITS2 amplicons using the WAVE microbial analysis system and peak analysis parameters for the Navigator Software 2.2.0 (Build 25) are listed.
(DOCX)

Table S2 **Average reciprocal Simpsońs indexes of diversity (1/D), standard deviations and P values of the compared groups of samples.**
(DOCX)
