# Supplementary material for: Gut Microbiota Patterns Associated with Colonization of Different Clostridium difficile Ribotypes
Source: PLoS One. 2013 Feb 28;8(2):e58005. doi: 10.1371/journal.pone.0058005 (PMC3585249; doi:10.1371/journal.pone.0058005)
Supplement: Table S1 — The DHPLC separation conditions and peak analysis parameters. Conditions for the separation of mixed species 16S rDNA or ITS2 amplicons using the WAVE microbial analysis system and peak analysis parameters for the Navigator Software 2.2.0 (Build 25) are listed. (DOCX) [file pone.0058005.s001.docx]

**Supplementary table S1. DHPLC separation conditions and peak analysis parameters.** Conditions for the separation of mixed species 16S rDNA or ITS2 amplicons using the WAVE microbial analysis system and peak analysis parameters for the Navigator Software 2.2.0 (Build 25) are listed.

| **Orgamism / Literature** | **DHPLC gradient** | | | | **Peak analysis parameters** | |
| --- | --- | --- | --- | --- | --- | --- |
| Bacteria /Doman *et al*., 2003 |  | Time (min) | % A^a^ | % B^b^ | **Peak detection window** | |
|  | Loading | 0 | 46 | 54 | Start time | 3.5 |
|  | Step 1 | 0.5 | 40 | 60 | Stop time | 11 |
|  | Step 2 | 2.5 | 39 | 61 | **Detection threshold** | |
|  | Step 3 | 4.5 | 38 | 62 | Absorbance | 0.05 |
|  | Step 4 | 8.5 | 37 | 63 | **Detection parameters** | |
|  | Step 5 | 10.5 | 36 | 64 | Global baseline order | 1 |
|  | Start clean | 10.6 | 46 | 54 | Bunch factor | 2 |
|  | Stop clean | 10.7 | 46 | 54 | Group separation | 0.5 |
|  | Start equilibrate | 10.8 | 46 | 54 | Noise factor | 4.4 |
|  | Stop equilibrate | 11.6 | 46 | 54 | Edge slope | 0.17 |
|  | The program was run at 62°C and at a flow rate of 0.35 ml/min. | | | | Peak rejection % | 10 |
|  |  | | | | Shoulder slope | 0.1 |

*(continued)*

| **Orgamism / Literature** | **DHPLC gradient** | | | | **Peak analysis parameters** | |
| --- | --- | --- | --- | --- | --- | --- |
| Fungi / Goldenberg *et al*., 2005 |  | Time (min) | % A^a^ | % B^b^ | **Peak detection window** | |
|  | Loading | 0 | 52 | 48 | Start time | 3 |
|  | Start gradient | 0.1 | 45 | 55 | Stop time | 9 |
|  | Stop gradient | 18.1 | 36 | 64 | **Detection threshold** | |
|  | Start clean | 18.2 | 52 | 48 | Absorbance | 0.05 |
|  | Stop clean | 18.3 | 52 | 48 | **Detection parameters** | |
|  | Start equilibrate | 18.4 | 52 | 48 | Global baseline order | 1 |
|  | Stop equilibrate | 19.9 | 52 | 48 | Bunch factor | 2.9 |
|  | The program was run at 60°C and at a flow rate of 0.5 ml/min. | | | | Group separation | 0.6 |
|  |  | | | | Noise factor | 2.6 |
|  |  | | | | Edge slope | 1.6 |
|  |  | | | | Peak rejection % | 48 |
|  |  | | | | Shoulder slope | 1 |

*(continued)*

| **Orgamism / Literature** | **DHPLC gradient** | | | | **Peak analysis parameters** | |
| --- | --- | --- | --- | --- | --- | --- |
| Archaea / This study |  | Time (min) | % A^a^ | % B^b^ | **Peak detection window** | |
|  | Loading | 0 | 47.6 | 52.4 | Start time | 1 |
|  | Start gradient | 0.5 | 42.6 | 57.4 | Stop time | 5.5 |
|  | Stop gradient | 5 | 33.6 | 66.4 | **Detection threshold** | |
|  | Start clean | 5.1 | 47.6 | 52.4 | Absorbance | 0.05 |
|  | Stop clean | 5.2 | 47.6 | 52.4 | **Detection parameters** | |
|  | Start equilibrate | 5.3 | 47.6 | 52.4 | Global baseline order | 1 |
|  | Stop equilibrate | 5.4 | 47.6 | 52.4 | Bunch factor | 2 |
|  | The program was run at 59.5°C and at a flow rate of 0.5 ml/min. | | | | Group separation | 0.6 |
|  |  | | | | Noise factor | 2.6 |
|  |  | | | | Edge slope | 1.6 |
|  |  | | | | Peak rejection % | 10 |
|  |  | | | | Shoulder slope | 0.4 |

^a^A, 0.1 M triethylammonium acetate; ^b^B, 0.1 M triethylammonium acetate in 25% acetonitrile
