# Supplementary material for: Gut Microbiota Patterns Associated with Colonization of Different Clostridium difficile Ribotypes
Source: PLoS One. 2013 Feb 28;8(2):e58005. doi: 10.1371/journal.pone.0058005 (PMC3585249; doi:10.1371/journal.pone.0058005)
Supplement: Table S2 — Average reciprocal Simpsońs indexes of diversity (1/D), standard deviations and P values of the compared groups of samples. (DOCX) [file pone.0058005.s002.docx]

**Supplementary table S2. Average reciprocal Simpson´s indexes of diversity (1/D), standard deviations and P values of the compared groups of samples.**

|  |  | **AVERAGE (1/D)^a^** | | **SD^b^** | |  |
| --- | --- | --- | --- | --- | --- | --- |
|  | **Sample set 1 vs. Sample set 2 ^a^** | **Sample set 1** | **Sample set 2** | **Sample set 1** | **Sample set 2** | **P-value^d^** |
| **BACTERIA** | **NEG-F (29)/Healthy (37)** | 6.2452 | 10.0328 | 2.6829 | 3.6591 | <0,0001 |
|  | **NEG-D (37)/Healthy (37)** | 6.0181 | 10.0328 | 3.0760 | 3.6591 | <0,0001 |
|  | **OTHER-F (17)/Healthy (37)** | 6.2529 | 10.0328 | 2.2877 | 3.6591 | 0.0003 |
|  | **POS-T (31)/Healthy (37)** | 8.3764 | 10.0328 | 8.2925 | 3.6591 | <0,0001 |
|  | **027-F (16)/Healthy (37)** | 4.4727 | 10.0328 | 1.6898 | 3.6591 | <0,0001 |
|  | **027-T (41)/Healthy (37)** | 5.3775 | 10.0328 | 2.4361 | 3.6591 | <0,0001 |
|  | **027-F (16)/NEG-F (29)** | 4.4727 | 6.2452 | 1.6898 | 2.6829 | 0.0214 |
|  | **027-T (41)/POS-T (31)** | 5.3775 | 8.3764 | 2.4361 | 8.2925 | 0.0315 |
|  | **027-F (16)/POS-F (17)** | 4.4727 | 6.2529 | 1.6898 | 2.2877 | 0.0167 |
|  | **POZ-T (31)/NEG-T (37)** | 8.3764 | 6.0181 | 8.2925 | 3.0760 | 0.1133 |
|  | **027-T (41)/NEG-T (37)** | 5.3775 | 6.0181 | 2.4361 | 3.0760 | 0.3116 |
|  | **NEG-F (29)/NEG-T (37)** | 6.2452 | 6.0181 | 2.6829 | 3.0760 | 0.7541 |
|  | **POZ-F (17)/NEG-F (29)** | 6.2529 | 6.2452 | 2.2877 | 2.6829 | 0.9922 |
| **FUNGI** | **NEG-F (29)/Healthy (37)** | 1.3564 | 1.3724 | 0.5635 | 0.5254 | 0.9130 |
|  | **NEG-D (37)/Healthy (37)** | 1.3531 | 1.3724 | 0.5193 | 0.5254 | 0.8821 |
|  | **OTHER-F (17)/Healthy (37)** | 1.2519 | 1.3724 | 0.3487 | 0.5254 | 0.4232 |
|  | **POS-T (31)/Healthy (37)** | 1.4373 | 1.3724 | 0.5203 | 0.5254 | 0.6739 |
|  | **027-F (16)/Healthy (37)** | 1.3368 | 1.3724 | 0.5761 | 0.5254 | 0.8413 |
|  | **027-T (41)/Healthy (37)** | 1.4515 | 1.3724 | 0.6058 | 0.5254 | 0.5705 |
|  | **027-F (16)/NEG-F (29)** | 1.3368 | 1.3564 | 0.5761 | 0.5635 | 0.9206 |
|  | **027-T (41)/POS-T (31)** | 1.4373 | 1.3531 | 0.5203 | 0.5193 | 0.5846 |
|  | **027-F (16)/POS-F (17)** | 1.4515 | 1.3531 | 0.6058 | 0.5193 | 0.4826 |
|  | **POZ-T (31)/NEG-T (37)** | 1.3564 | 1.3531 | 0.5635 | 0.5193 | 0.9818 |
|  | **027-T (41)/NEG-T (37)** | 1.2519 | 1.3564 | 0.3487 | 0.5635 | 0.5239 |

^a^Arithmetic mean.

^b^Standard deviation.

^c^ The compared sample sets and the number of samples in each set is specified. NEG-F: *C. difficile* negative/formed stool; NEG-D: *C. difficile* negative/diarrhoea; POS-F: *C. difficile* non 027 ribotype/formed stool; POS-D: *C. difficile* non 027 ribotype/diarrhoea; 027-F: *C. difficile* 027 ribotype/formed stool; 027-D: *C. difficile* 027 ribotype/diarrhoea; Healthy: healthy donors.

^d^ The two-tailed P-value.
